# Inherited epidermolysis bullosa

**DOI:** 10.1186/1750-1172-5-12

**Published:** 2010-05-28

**Authors:** Jo-David Fine

**Affiliations:** 1 Departments of Medicine (Dermatology) and Pediatrics Vanderbilt University School of Medicine, and Head, National Epidermolysis Bullosa Registry Nashville, TN, USA

## Abstract

Inherited epidermolysis bullosa (EB) encompasses a number of disorders characterized by recurrent blister formation as the result of structural fragility within the skin and selected other tissues. All types and subtypes of EB are rare; the overall incidence and prevalence of the disease within the United States is approximately 19 per one million live births and 8 per one million population, respectively. Clinical manifestations range widely, from localized blistering of the hands and feet to generalized blistering of the skin and oral cavity, and injury to many internal organs. Each EB subtype is known to arise from mutations within the genes encoding for several different proteins, each of which is intimately involved in the maintenance of keratinocyte structural stability or adhesion of the keratinocyte to the underlying dermis. EB is best diagnosed and subclassified by the collective findings obtained via detailed personal and family history, in concert with the results of immunofluorescence antigenic mapping, transmission electron microscopy, and in some cases, by DNA analysis. Optimal patient management requires a multidisciplinary approach, and revolves around the protection of susceptible tissues against trauma, use of sophisticated wound care dressings, aggressive nutritional support, and early medical or surgical interventions to correct whenever possible the extracutaneous complications. Prognosis varies considerably and is based on both EB subtype and the overall health of the patient.

## 

Disease name: epidermolysis bullosa

Synonyms: see Table 1 and [1]

**Table 1:** EB synonyms

Name	Synonym(s)
Inherited EB	EB hereditaria
EB simplex, localized	EB simplex, Weber-Cockayne; EB simplex of palms and soles
EB simplex, Dowling-Meara	EB (simplex) herpetiformis
EB simplex, generalized non-Dowling-Meara	EB simplex, Koebner EB simplex, generalized other
Junctional EB	EB atrophicans
JEB, Herlitz	JEB generalisata gravis
JEB, non-Herlitz	JEB generalisata mitis
Dystrophic EB (DEB)	EB dystrophica
Dominant dystrophic EB (DDEB)	DDEB, Pasini and Cockayne-Touraine variants
Recessive dystrophic EB (RDEB), severe generalized	RDEB, Hallopeau-Siemens; RDEB generalisata gravis
RDEB, generalized other	RDEB, non-Hallopeau-Siemens; RDEB generalisata mitis
EB with congenital localized atrophy of skin	Bart's syndrome

## Definition

Inherited epidermolysis bullosa (EB) encompasses over 30 phenotypically or genotypically distinct entities which share as a common feature mechanical fragility of epithelial lined or surfaced tissues, most notably the skin [2]. A characteristic feature of all types of EB is the presence of recurrent blistering or erosions, the result of even minor traction to these tissues.

## Classification

In general, patients with EB are classified and subclassified based on the ultrastructural level within which blisters develop within the skin (Table 2), mode of inheritance, and combinations of clinical, electron microscopic (Table 3), immunohistochemical (Table 4), and genotypic features. Each of the major EB subtypes is discussed in great detail in the 2008 consensus report on diagnosis and classification [3], which was based on the recommendations of an international panel of EB experts, superceding two previously recommended classification schemes [4,5].

**Table 2:** Level of blister formation in each major EB type

Major EB Types	Level of Blister Formation
EB simplex	Intraepidermal
Junctional EB	Intra-lamina lucida
Dystrophic EB	Sub-lamina densa
Kindler syndrome	Multiple levels (intra-lamina lucida and sub-lamina densa)

**Table 3:** Transmission electron microscopy findings among selected EB subtypes

EB type	Major EB subtype	Level of cleavage	Associated ultrastructural findings
EB simplex (suprabasal)	EB simplex superficialis	subcorneal	---
	lethal acantholytic EB	suprabasal	acantholysis; perinuclear retraction of keratin filaments
	EBS, plakophilin deficiency	mid-epidermis	perinuclear retraction of keratin filaments; small suprabasal desmosomes
EBS (basal)	EBS, localized	basal keratinocyte	---
	EBS, Dowling-Meara	basal keratinocyte	clumped keratin filaments
	EBS, generalized other	basal keratinocyte	---
	EBS, autosomal recessive	basal keratinocyte	absent or reduced keratin filaments within basal keratinocytes
Junctional EB	JEB, Herlitz	intra-lamina lucida	markedly reduced or absent hemidesmosomes; absent subbasal dense plates; absent anchoring filaments
	JEB, non-Herlitz	intra-lamina lucida	hemidesmosomes may be normal or reduced in size and number
	JEB with pyloric atresia	intra-lamina lucida	small hemidesmosome plaques with attenuated subbasal dense plates
Dominant dystrophic EB	DDEB, generalized	sub-lamina densa	normal or reduced numbers of anchoring fibrils
	DDEB, bullous dermolysis of the newborn	sub-lamina densa	electron-dense stellate shaped bodies within basal keratinocytes; reduced numbers of anchoring fibrils
Recessive dystrophic EB	RDEB, severe generalized	sub-lamina densa	absent or rudimentary appearing anchoring fibrils
	RDEB, generalized other (generalized mitis)	sub-lamina densa	reduced or rudimentary appearing anchoring fibrils

**Table 4:** Diagnostically useful differences in antigenic staining in selected EB subtypes

Common antigens	Abnormal staining in	Typical pattern of staining
Keratin 5	---	normal filamentous network within keratinocytes
Keratin 14	EBS, autosomal recessive	absent or markedly reduced staining of filaments within keratinocytes
Laminin-332 (laminin-5)	JEB-H	absent or markedly reduced staining along the DEJ
	JEB-nH *	reduced staining along the DEJ
Type XVII collagen	JEB-nH *	reduced or absent staining along the DEJ
Type VII collagen	RDEB, severe generalized	usually absent staining along the DEJ
	RDEB, generalized other	reduced staining along the DEJ
	RDEB, inversa	variable staining along the DEJ
	Dystrophic EB-BDN (only during periods of active blistering)	granular staining within basal and lower suprabasal keratinocytes; absent or markedly reduced staining along the DEJ
Plectin	EBS with muscular dystrophy; EBS with pyloric atresia; EBS-Ogna	absent or reduced staining along the DEJ; absent or reduced staining along the DEJ; reduced staining along the DEJ
α6β4 integrin	JEB with pyloric atresia; EBS with pyloric atresia	absent or reduced staining along the DEJ; absent or reduced staining along the DEJ
Kindlin-1	Kindler syndrome	absent or reduced staining along the DEJ

DEJ = dermoepidermal junction

* The majority of patients with JEB-nH have mutations in one of the three genes encoding for laminin-332, rather than within the gene for type XVII collagen. Of note, there are usually no phenotypic differences in these two antigenically distinct JEB-nH groups.

Modified from Fine J-D et al, 2008 (reference [3]); refer to that reference for less frequent findings

There are four major types of inherited EB: EB simplex (EBS), junctional EB (JEB), dystrophic EB (DEB), and Kindler syndrome [6]. These differ not only phenotypically and genotypically but more importantly by the site of ultrastructural disruption or cleavage. Intraepidermal blistering is the hallmark feature of EB simplex. EB simplex patients are then further subclassified, based on whether blisters arise within the basal (i.e., lowermost) or suprabasal (upper) layers of the epidermis [3]. In contrast, JEB and DEB patients develop their blisters within the lamina lucida and sub-lamina densa of the skin basement membrane zone ("dermoepidermal junction"), respectively. In Kindler syndrome, multiple cleavage planes may be seen within the same sample of skin [7]. Table 5 lists each of the major EB types and subtypes, as recognized in the latest consensus report. As reported in that publication, the major additions to previous classification schemes have been (1) the subdivision of EBS patients into both basal and suprabasal subtypes, to include three rare mechanobullous disorders having blister formation within the upper epidermis; (2) the addition of a fourth major EB type, Kindler syndrome, which had previously been considered as a poikilodermatous photosensitivity disease; and (3) inclusion of the laryngo-onycho-cutaneous syndrome (LOC; previously called Shabbir's syndrome) [8,9], given its shared molecular target with that of JEB [10].

**Table 5:** Major EB subtypes and their targeted proteins (per the 2008 international consensus report [3])

Major EB Type	Major EB Subtypes	Targeted Protein(s)
EB simplex (EBS)	suprabasal subtypes	
	lethal acantholytic EBS	desmoplakin
	plakophilin-1 deficiency	plakophilin-1
	EBS superficialis (EBSS)	?
		
	basal subtypes	
	EBS, localized (EBS-loc)	K5, K14
	EBS, Dowling-Meara (EBS-DM)	K5, K14
	EBS, other generalized (EBS,-gen nDM)	K5, K14
	EBS with mottled pigmentation (EBS-MP)	K5
	EBS with muscular dystrophy (EBS-MD)	plectin
	EBS with pyloric atresia (EBS-PA)	plectin; α6β4 integrin
	EBS, autosomal recessive (EBS-AR)	K14
	EBS, Ogna (EBS-Og)	plectin
	EBS, migratory circinate (EBS-migr)	K5
		
Junctional EB (JEB)	JEB, Herlitz (JEB-H)	laminin-332
	JEB, generalized non-Herlitz (JEB-nH gen)	laminin-332; type XVII collagen
	JEB, localized non-Herlitz (JEB-nH loc)	type XVII collagen
	JEB with pyloric atresia (JEB-PA)	α6β4 integrin
	JEB, inversa (JEB-I)	laminin-332
	JEB, late onset (JEB-lo)	?
	LOC syndrome	laminin-332 α3 chain
		
Dominant dystrophic EB (DDEB)	DDEB, generalized (DDEB-gen)	type VII collagen
	DDEB, acral (DDEB-ac)	type VII collagen
	DDEB, pretibial (DDEB-Pt)	type VII collagen
	DDEB, pruriginosa (DDEB-Pr)	type VII collagen
	DDEB, nails only (DDEB-na)	type VII collagen
	DDEB, bullous dermolysis of newborn (DDEB-BDN)	type VII collagen
		
Recessive dystrophic EB (RDEB)	RDEB, severe generalized (RDEB-sev gen)	type VII collagen
	RDEB, generalized other (RDEB, generalized mitis (RDEB-O)	type VII collagen
	RDEB, inversa (RDEB-I)	type VII collagen
	RDEB, pretibial (RDEB-Pt)	type VII collagen
	RDEB, pruriginosa (RDEB-Pr)	type VII collagen
	RDEB, centripetalis (RDEB-Ce)	type VII collagen
	RDEB, bullous dermolysis of newborn (RDEB-BDN)	type VII collagen
		
Kindler syndrome		kindlin-1

Modified from Fine JD et al (reference [3])

## Epidemiology

Estimates of prevalence and incidence of EB have been attempted by different sampling techniques in a number of populations worldwide, but the most rigorously obtained ones are derived from the National EB Registry (NEBR), a cross-sectional and longitudinal epidemiological study of EB patients across the entire continental United States. Over its 16 years (1986-2002) of formal funding by the National Institutes of Health, nearly 3,300 EB patients were identified, enrolled, classified, clinically characterized, and followed for outcomes. Among this robust study population, the prevalence and incidence of EB was estimated as approximately 8 per one million population and 19 per one million live births, in 1990 and 1986-1990, respectively [11-13]. These data were then used to estimate carrier frequencies for EB within the United States [14]. The NEBR prevalence and incidence estimates are very similar to most of those reported elsewhere (the largest cohort of which is the Italian EB Registry [15]) [16-18], many of which used less rigorous epidemiological sampling methods. The overall consistency of these data in different parts of the world suggests that the epidemiological data that have been derived by the NEBR can indeed be generalized worldwide. Of note, a greater prevalence of EBS has been reported from Scotland [19]. It is unclear whether this reflects possible greater accessibility of EBS patients for identification and recruitment in the Scottish Registry or the presence of some underlying genetic differences which might distinguish the Scottish EB population from those in other geographic regions. For example, the American NEBR data on EBS are based on the actual findings of the NEBR study population [11]. Revised estimates of both prevalence and incidence in the United States, based on assumptions of incomplete capture of EBS cases, have been published in greater detail in the peer-reviewed monograph which was based on the NEBR database [11]. Given how relatively mild the disease activity usually is in localized EBS, it is indeed possible that our prevalence and incidence data on American EBS patients may be an underestimate if our enrollment of EBS patients had been discordantly low, although our overall data so closely match those from Italy that this hypothesis is probably incorrect.

An important demographic finding among the NEBR study population was the lack of any EB type or subtype predilection by gender or ethnicity. In particular, when allowances were made for ethnic differences in access to specialty care within the United States, the overall distribution of NEBR subjects closely resembled that of the general American population [20].

As anticipated from previous anecdotal experiences worldwide, the majority of EB patients in the NEBR cohort had some type of EB simplex [11]. Of these, the majority had the localized subtype. As also expected, the majority of patients with generalized junctional EB (JEB) had the less severe (non-Herlitz) subtype, and the majority of patients with generalized recessive dystrophic EB (RDEB) had the clinically less severe (non-Hallopeau-Siemens) subtype of this disease.

## Clinical description

### General considerations [2]

**Figure 1:**
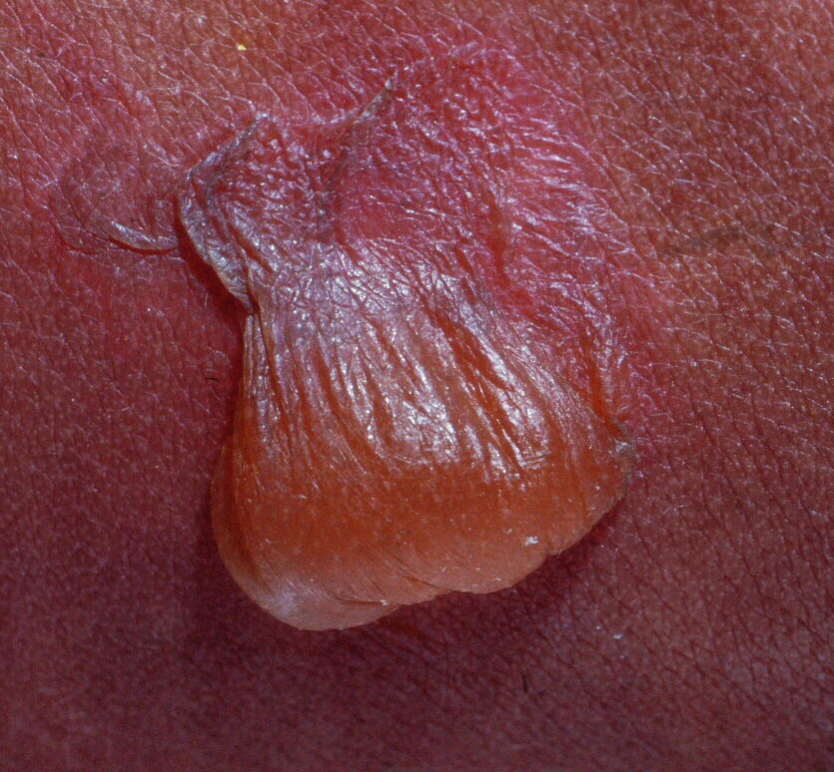
**A typical noninflammatory blister arising in the skin of a patient with EB**.

**Figure 2:**
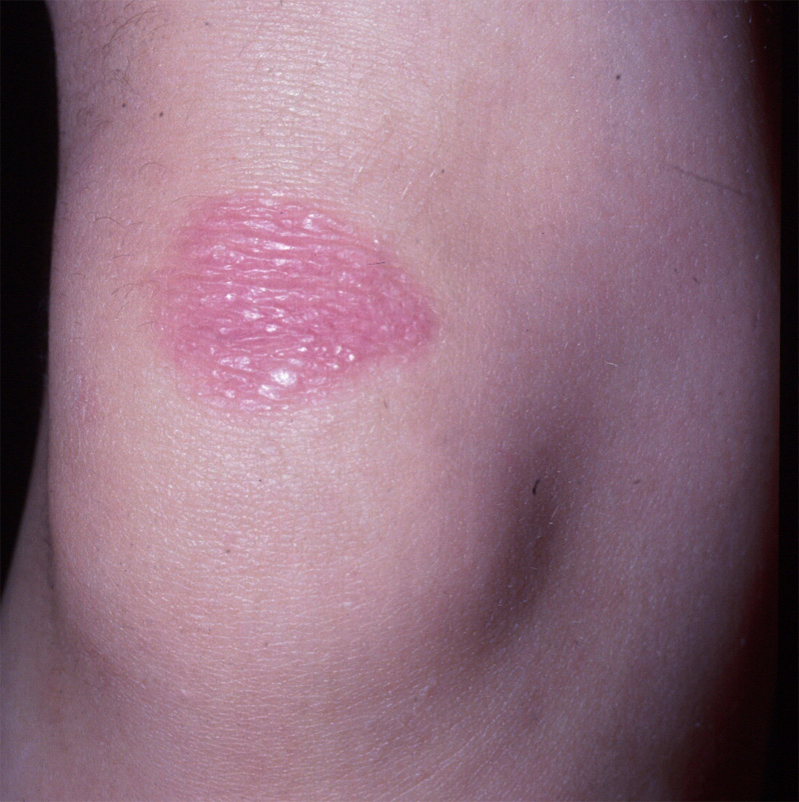
**Milia arising within an erythematous patch on the knee of a patient with DDEB**.

The hallmark cutaneous features of inherited EB, in addition to mechanically fragile skin and easy inducibility of blisters (Figure 1) or erosions, include some or all of the following: milia (tiny firm white papules, resembling cysts or pustules) (Figure 2), nail dystrophy or absence, and scarring (usually atrophic). Additionally useful findings, if present, include exuberant granulation tissue (periorificial; axillary vaults; nape of the neck; lumbosacral spine; periungual and proximal nail folds), localized or confluent keratoderma of the palms and soles, and dyspigmentation (postinflammatory hypo- or hyperpigmentation; mottled or reticulate hyperpigmentation). Infrequently seen and extremely nonspecific cutaneous findings include decreased or absent hair, albopapuloid lesions (flesh-colored or hypopigmented papules, usually arising on the lower trunk), and hypo- or hyperhidrosis [21].

Several factors must be considered when attempting to use cutaneous findings as surrogate diagnostic markers. First, the presence or absence of one or more findings may be age-dependent [22]. That is, not all of these skin findings are necessarily seen in neonates or infants. For example, scarring, nail dystrophy, milia, and exuberant granulation tissue may develop only after several months or even years of life. As such, their absence cannot be reliably used for diagnosis during that window of time (i.e., early infancy) when classification and subclassification are most needed [22]. Similarly, exuberant granulation tissue, the most pathognomonic skin finding in EB, which is seen almost exclusively in the Herlitz subtype of generalized JEB, may completely resolve spontaneously in rare patients during adulthood. Second, some findings (such as albopapuloid lesions or aplasia cutis) may arise in more than one EB type or subtype, making them insufficiently specific to be reliably used as diagnostic tools [21]. Indeed, when sensitivity and specificity analyses were performed on the robust database of the NEBR, the only cutaneous finding reaching 90% in both sensitivity and specificity as a surrogate diagnostic marker (even when up to three findings were considered in combination) was exuberant granulation tissue, emphasizing the inherent risks associated with relying too heavily solely on the presence or absence of EB-associated skin findings [22].

All types and subtypes of inherited EB are associated with mechanically fragile skin. This can be induced by light lateral or rotary traction to the skin. In general, skin from patients with JEB and RDEB is much more fragile than that of EBS patients. Although most EB patients usually present with intact blisters, erosions may instead be the overriding finding in patients having one of the more superficial (i.e., suprabasal) EBS subtypes [3]. Skin fragility in EB is characteristically worsened in warm weather or warm living environments; hence the value of air-conditioning for families with affected children. The one exception to this observation, which is seen in only a subset of EBS-DM patients, is a temporary reduction of blistering during periods of high fever. Of practical importance, a history of seasonally dependent (i.e., summertime) blistering is more often elicited in EB patients having milder or more localized types or subtypes of the disease, especially those with EBS, since the most severely affected patients (particularly those with generalized JEB and RDEB) have such continuous generalized blistering that any influence by temperature is clinically negligible.

Potentially any extracutaneous tissue which is lined or surfaced by epithelium may be injured in EB [23-25]. In general, the more severe and widely distributed the blistering on the skin is, the more likely it is for multiple extracutaneous sites to also become involved. As is the case with cutaneous findings, these extracutaneous complications are also age-dependent, with time of onset and cumulative risk of occurrence highly dependent on the EB subtype that is present. Examples of the cumulative risks for the most important extracutaneous complications will be reviewed in detail elsewhere in the context of specific EB subtypes. As will also be discussed subsequently, other non-epithelial organs or tissues may also be involved in selected EB subtypes. For example, enamel hypoplasia [26] is seen exclusively in all subtypes of JEB (and is therefore a highly useful diagnostic finding), and muscular dystrophy (either congenital or late onset) may accompany one specific subtype of EBS. Some other organs, for example, the heart and kidney, may also become secondarily injured in severely affected EB patients.

When counseling a family with an affected child, it is important to realize that considerable variability in the frequency and severity of extracutaneous complications may be seen within not only a single major EB subtype but even within a kindred. The risk and time of onset of occurrence of these complications may vary considerably from one EB subtype to another. Some EB types and subtypes are particularly at risk for premature death from one or more causes. Finally, genotype-phenotype correlation, with the exception of EBS, is rather weak [3].

### EB simplex

As discussed in much greater detail within the 2008 consensus report [3], there is a wide range of cutaneous findings among the many EBS subtypes. With only three rare exceptions (Table 5), blisters arise within the basal layer of the epidermis in patients with EBS. Onset of disease activity in EBS is usually at or shortly after birth, although patients with localized EBS may not develop blistering until late childhood or even early adulthood. As a general rule, far less scarring, milia formation, and nail dystrophy are seen in EBS, as compared to JEB and DEB, although even the combination of all three of these clinical findings, when used as a diagnostic test to distinguish EBS from all other EB types, failed to provide both sensitivity and specificity of > 90% [21].

**Figure 3:**
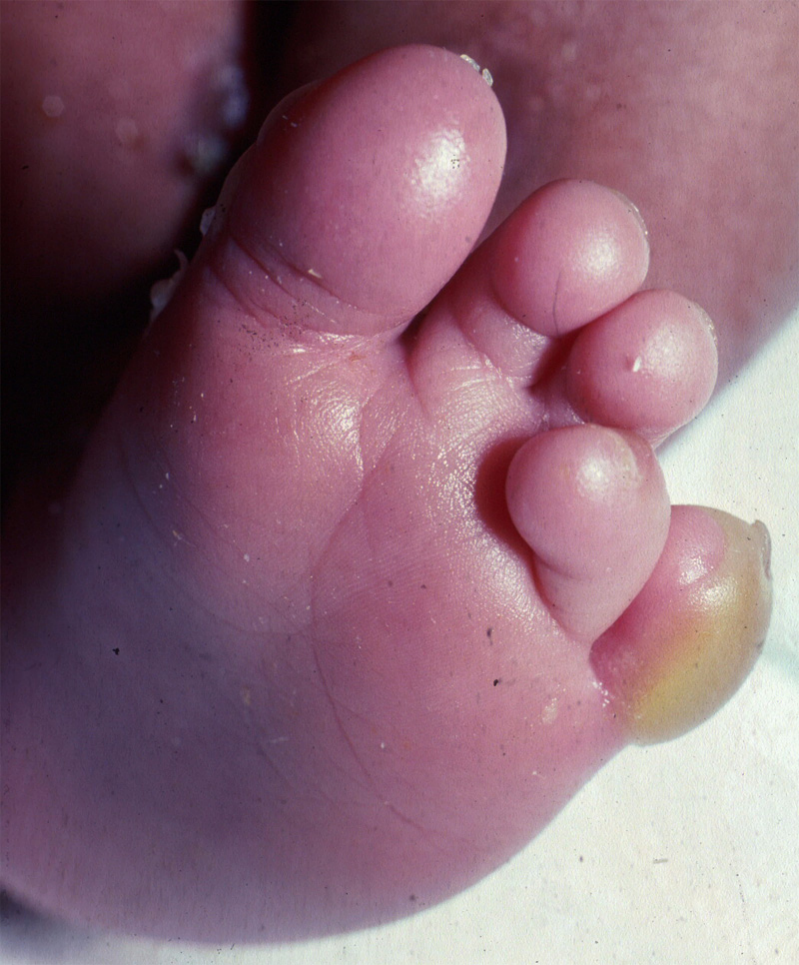
**The blistered foot of an infant with localized EBS**.

The most common type of EB, as well as the most common subtype of EBS, is *localized EBS*, formerly known as Weber-Cockayne disease. Among the National EB Registry population, half of all participants had EBS; of these, two-thirds had localized EBS [20]. The usual distribution of blisters in these patients is on the palms and soles (Figure 3), although any other skin surface may also blister if sufficiently traumatized. Milia, scarring, and nail dystrophy are uncommon to rare skin findings in all forms of EBS, with the lowest frequency noted in localized EBS. The only common extracutaneous finding in localized EBS, localized intraoral erosions or blisters, tends to be asymptomatic, occurs in about one-third of these patients, and usually is seen only during infancy [27,28].

**Figure 4:**
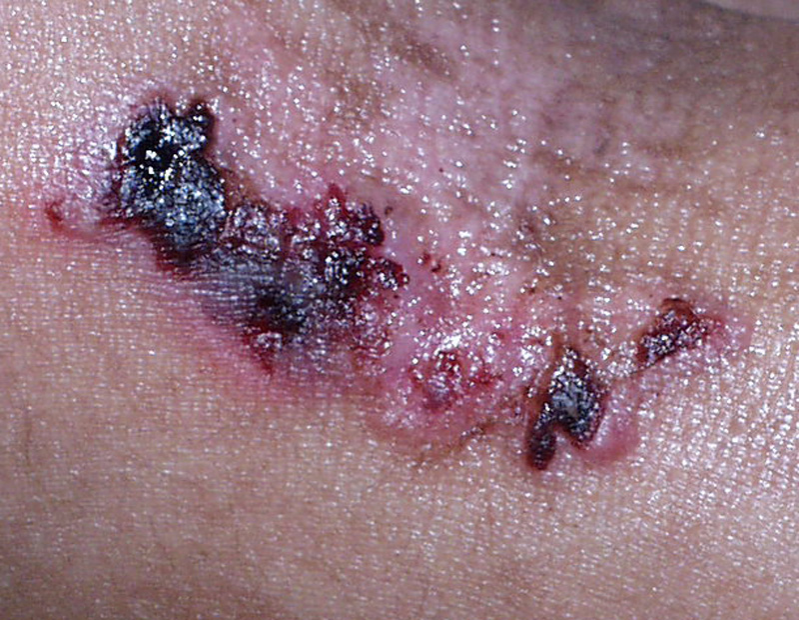
**Circinate grouping of blisters arising on the skin of a patient with the Dowling-Meara variant of generalized EBS**.

There are several subtypes of more generalized EBS. The most noteworthy one, *EBS-Dowling Meara *(DM) [29], is frequently associated with marked morbidity and, in a minority of patients, may result in death during early infancy. Its hallmark feature is the presence of intact vesicles or small blisters in grouped or arcuate configuration (Figure 4). Such herpes simplex-like clustering of lesions explains why this entity was originally named EB herpetiformis, although there is no true association between this disease and either herpetic infection or the autoimmune bullous disease, dermatitis herpetiformis. By late childhood, most patients with EBS-DM develop confluent thickening and hyperkeratosis ("keratoderma") of the palms and soles which may partially resolve in some patients during mid- to late-adulthood. Although not a universal finding, some patients with EBS-DM may improve when febrile, which is paradoxical, since warmer weather exacerbates disease activity in all EB patients. The reason for such a phenomenon is unknown. The most notable extracutaneous complication in EBS, and one that is seen in only rare patients with EBS-DM, is tracheolaryngeal compromise, mimicking that which arises in both major subtypes of JEB [30,31]. There is also a markedly increased risk of developing basal cell carcinomas by mid-adulthood (cumulative risk of 44% by age 55) [32], a finding seen in EB only among patients with EBS-DM.

Another subtype of generalized EBS ("*EBS generalized other*" or *non-Dowling-Meara generalized EBS*), formerly named *EBS-Koebner (EBS-K)*, is characterized by non-herpetiform blisters and erosions arising on any skin surface [33]. Of note, these blisters tend to spare the palms and soles, distinguishing them from patients with localized EBS. The frequency of milia, scarring, and nail dystrophy is intermediate between that of localized EBS and EBS-DM, and extracutaneous findings, other than occasional intraoral blistering, are rare. Given the considerable overlap between EBS-K and localized EBS within some kindreds, some experts prefer to group both subtypes together.

Other clinically striking but rare basal EB subtypes include *EBS with mottled pigmentation (EBS-MP) *[34,35], *EBS with muscular dystrophy (EBS-MD) *[36,37], *autosomal recessive EBS *[38], *EBS with circinate migratory blistering *[39], and *EBS-Ogna *[40,41], the latter of which is associated with easy bruisability, hemorrhagic blistering, and marked nail deformity (onychogryphosis) [3]. From a prognostic point of view, immunohistochemical recognition of EBS-MD in infancy is particularly important, since in some patients the associated muscular dystrophy may not become apparent until later in childhood or early adulthood.

### Junctional EB

**Figure 5:**
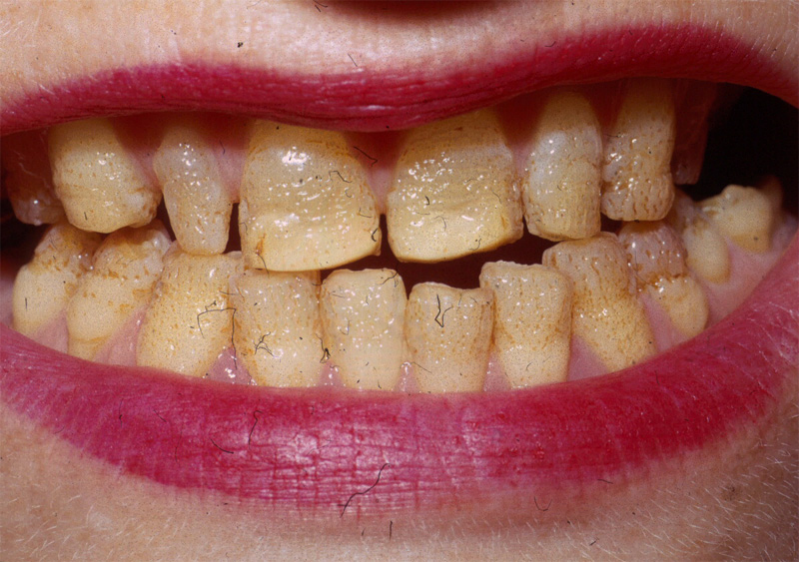
**Rather profound enamel pitting in a patient with JEB**.

There is only one clinical finding that is characteristic of all subtypes of JEB -- the presence of enamel hypoplasia, manifested as localized or more extensive thimble-like pitting of some or all of the tooth surfaces (Figure 5) [26,42]. It is therefore an extremely useful clinical finding, although it cannot be used as a diagnostic tool until after the primary teeth have erupted.

**Figure 6:**
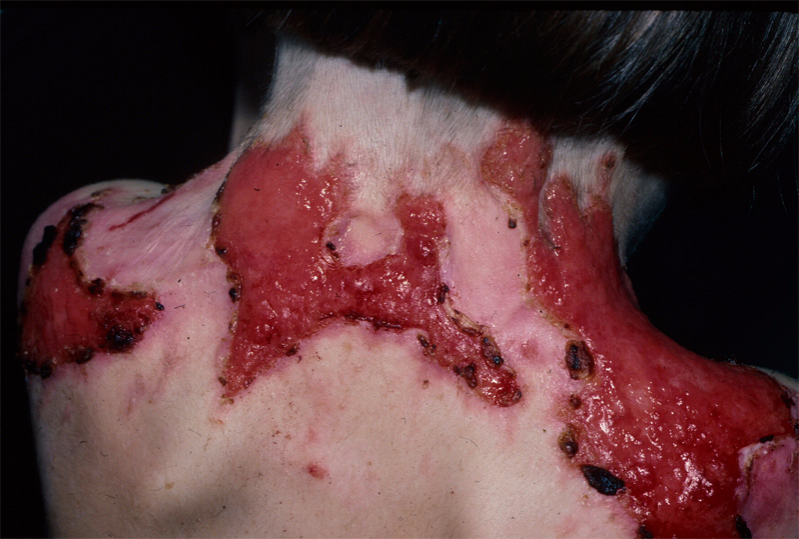
**Exuberant granulation tissue arising on the nape of the neck of a child with Herlitz JEB**.

There are two major JEB subtypes. The more severe one, *JEB-Herlitz (JEB-H)*, is present at birth and involves all skin surfaces [43]. Approximately 20% of all JEB patients within the United States have this phenotype [11]. An essentially pathognomonic finding is exuberant granulation tissue (Figure 6), which usually arises within the first several months to one to two years of life. This may involve not only the skin but also the upper airway. Moderate to severe intraoral blistering is invariably present, with some eventual narrowing of the opening of the mouth ("microstomia") and reduced extension of the tongue ("ankyloglossia"), although these findings are not as pronounced as is observed in severe generalized RDEB [27]. Rather profound growth retardation and multifactorial anemia are the norms in JEB-H [25]. Many other organs may be involved, including but not restricted to the esophagus (strictures) [44], external eye (corneal blisters, erosions, and scarring; ectropion formation) [45], upper airway (strictures and occlusion) [30], and genitourinary tract [46]. Among JEB-H children, for example, the cumulative risk of laryngeal stenosis or stricture is 40% by age 6 [30]. The highest risk of infant mortality among EB neonates, infants, and young children occurs in JEB-H, and is most often the result of sepsis, failure to thrive, or tracheolaryngeal obstruction (the latter usually secondary to severe progressive airway stenosis within or above the level of the vocal cords) [47]. Indeed, as of January 1, 2002, over half of all JEB-H patients enrolled in the NEBR had died within the first two years of life. Squamous cell carcinomas may also arise in a minority of JEB-H patients (cumulative risk of 18% by age 25) [32].

The most common JEB subtype, comprising nearly 80% of the American JEB population, is *non-Herlitz JEB (JEB-nH)*, a generalized disorder characterized by the presence of blistering, atrophic scarring, and nail dystrophy or absence. It, too, is usually clinically apparent at birth. Postinflammatory hypopigmentation or depigmentation may be striking in some JEB-nH patients. A prominent feature described among the original Tyrolean kindred was scarring alopecia of the scalp [48], although experience in other populations has shown that this is not a universal finding among JEB-nH patients. The cumulative risk of upper airway occlusion in JEB-nH (13% by age 9) is lower than that observed in JEB-H [30] although the risk of death among those JEB-nH patients with this complication is essentially the same. The frequencies of other extracutaneous complications, including severe anemia and growth retardation, however, are far lower among JEB-nH than JEB-H patients, as is the risk of premature death from non-airway-related complications [47].

A rare but clinically important JEB subtype, *inverse JEB*, is associated with rather severe blistering and erosions confined to intertriginous skin sites, esophagus, and vagina [3].

*JEB with pyloric atresia *presents with generalized blistering at birth and congenital atresia of the pylorus (and rarely of other portions of the gastrointestinal tract) [3,49]. This latter disorder is associated with a significant risk of congenital anomalies of the genitourinary tract and infantile or neonatal death. It should be noted that rare patients with identical phenotypes have been shown to have intraepidermal rather than intra-lamina lucida blister formation, necessitating their inclusion among the rarer subtypes of EBS rather than JEB.

The *LOC syndrome *is characterized by localized blistering and scarring, particularly on the face and neck, in association with upper airway disease activity and nail abnormalities [8,9]. Characteristic cutaneous findings are erosions and granulation tissue. The conjunctiva is involved and enamel hypoplasia is present.

### Dystrophic EB

Dystrophic EB (DEB) is separated into two major types, based on the mode of transmission (autosomal dominant versus autosomal recessive). As noted in Table 5, DEB patients are further subdivided by clinical phenotype and severity of disease [3].

#### Dominant dystrophic EB

**Figure 7:**
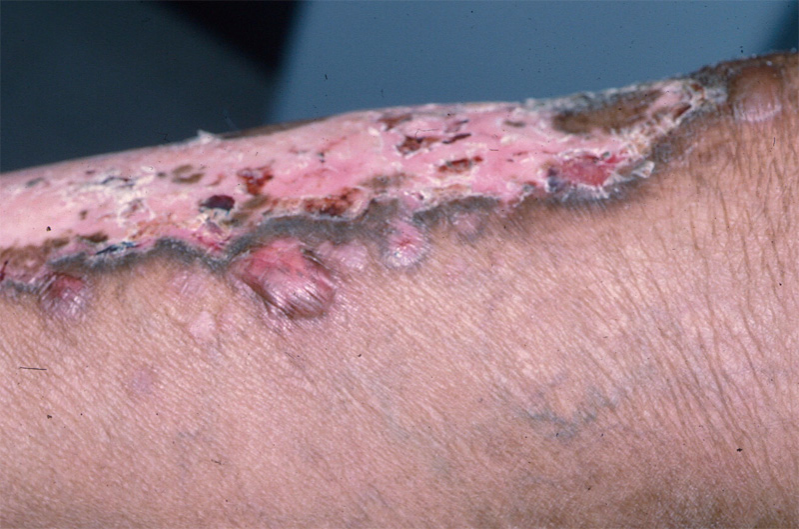
**Atrophic scarring and postinflammatory hypopigmentation on the extremity of a patient with DDEB**.

**Figure 8:**
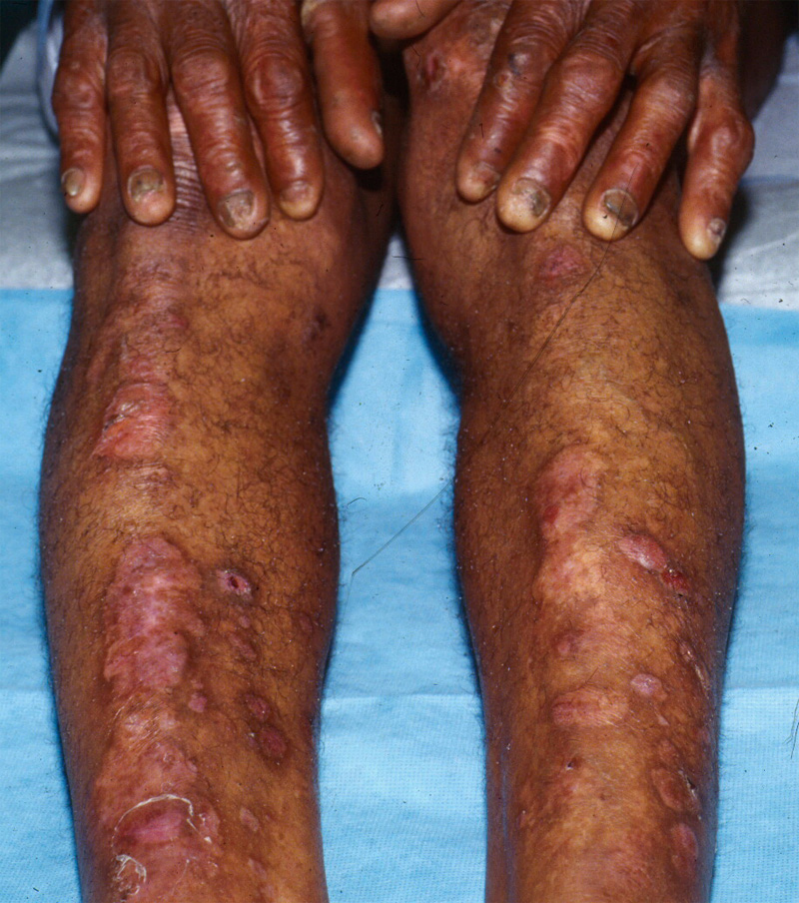
**Hypertrophic scarring in a patient with generalized DDEB**.

**Figure 9:**
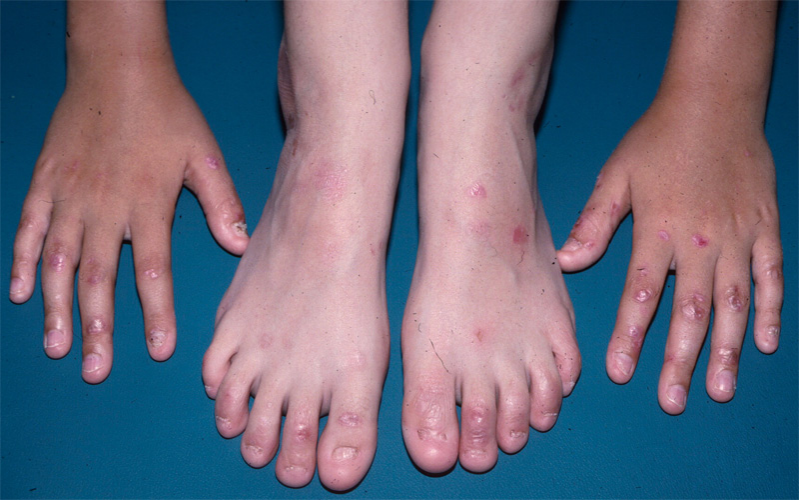
**Dystrophy of all twenty nails in a patient with DDEB**.

By convention, all patients with *generalized dominant DEB (DDEB) *are now grouped together. Although, in the past, two DDEB subtypes, Pasini [50] and Cockayne-Touraine DDEB [51], were considered to be different diseases, more recent studies have failed to confirm either the specificity of albopapuloid lesions or differences in genotype between these two putatively distinct DDEB subtypes [3]. The prototypic DDEB patient has generalized blistering at birth which, with time, is associated with mila, atrophic (or less commonly, hypertrophic) scarring (Figures 7 and 8), and nail dystrophy (Figure 9). Recurrent esophageal blistering and erosions, leading to progressive dysphagia secondary to esophageal stricture formation, is common among these patients [44]. In contrast to severe generalized RDEB and JEB-H, however, failure to thrive, growth retardation, severe anemia, early infant mortality, and risk of squamous cell carcinoma are not characteristic features of DDEB [24,25,47].

There are a number of less common DDEB subtypes, as listed in Table 5. A rare localized variant, *acral DDEB*, is characterized by cutaneous involvement confined primarily to the hands and feet. Of note, pseudosyndactyly is not a feature of this entity.

*Pretibial DDEB*, as the name implies, almost exclusively involves the anterior lower legs [52,53]. Individual lesions, which tend to be papular or plaque-like, are oftentimes somewhat violaceous, suggesting the clinical diagnosis of lichen planus. Bullae and scarring are also present. Dystrophy of both fingernails and toenails is characteristic; in contrast to lichen planus, these nail changes do not include pterygium formation.

*DDEB pruriginosa *is a more generalized subtype of DDEB which is characterized by severe, if not intractable, pruritus [54]. *Bullous dermolysis of the newborn (BDN) *is a subtype of dystrophic EB which is usually transmitted in an autosomal dominant manner [55]. It, like other generalized forms of EB, presents at or shortly after birth and may be accompanied by focal atrophic scarring. In contrast to all other types and subtypes of EB, disease activity usually ceases within the first 6 to 24 months of life.

**Figure 10:**
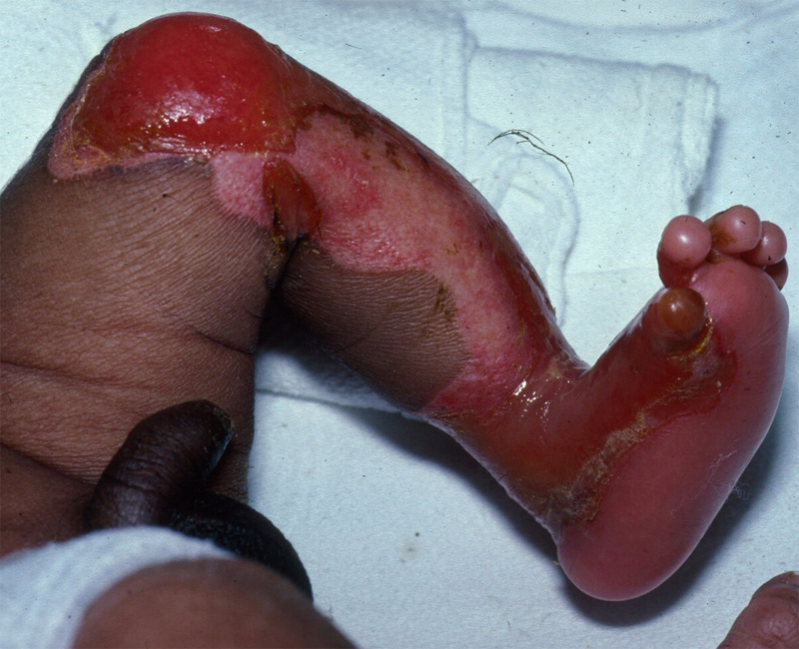
**Congenital absence of skin on the leg of a neonate with Bart's syndrome**.

A rare finding associated with EB and arising on one or more extremities, congenital localized absence of skin (CLAS) (Figure 10), was originally observed within a large kindred with autosomal dominant transmission of blistering; this family was later proven to have DDEB [56,57]. Referred to as *Bart's syndrome *and once believed to be a distinct entity, it is now known that CLAS may arise in EBS, JEB, and RDEB, as well as in DDEB. As such, the eponym is no longer used and the condition is no longer deemed to be a separate EB subtype [5].

#### Recessive dystrophic EB

**Figure 11:**
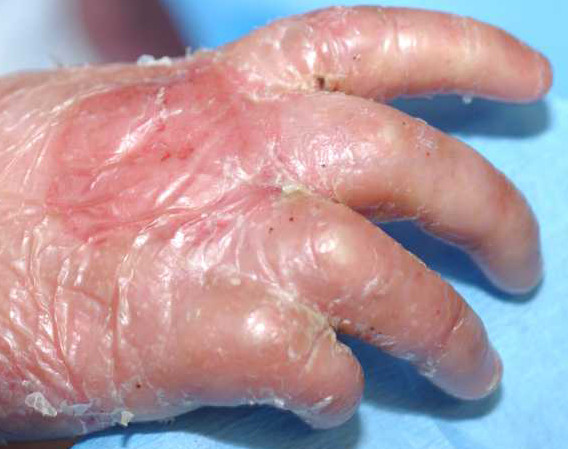
**Partial mitten deformity of the hand of a child with severe generalized RDEB**.

**Figure 12:**
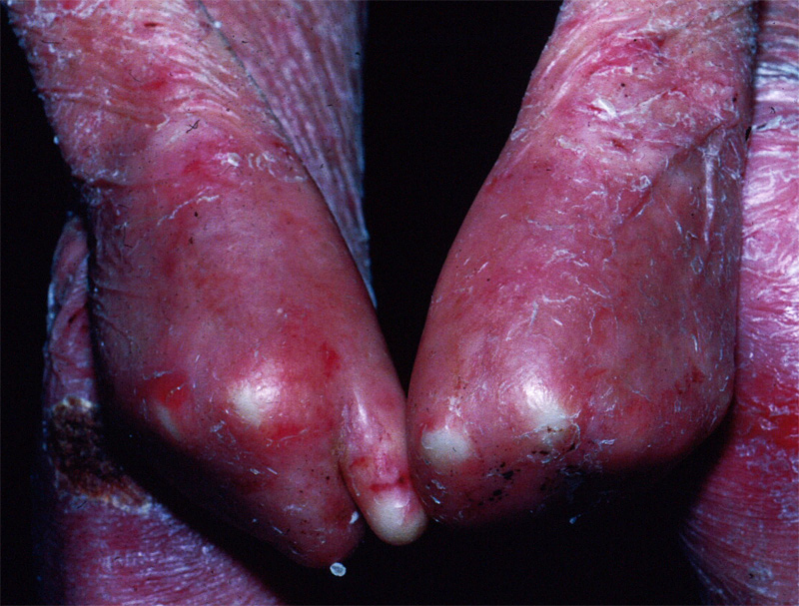
**Complete mutilating deformities of the hands of a young adult with severe generalized RDEB**.

There are three main subtypes of RDEB *-- severe generalized RDEB *(formerly named *Hallopeau-Siemens RDEB*), *non-Hallopeau-Siemens RDEB*, and *inverse RDEB*. Each has its onset at birth. The most severe subtype, severe generalized RDEB, is clearly one of the most devastating multiorgan genetically transmitted diseases of mankind. Prototypic findings include generalized blistering at birth, progressive and oftentimes mutilating scarring of the skin, corneal blisters or scarring [45], profound growth retardation [44], multifactorial anemia, failure to thrive (less common than in JEB-H), esophageal strictures [44], and debilitating hand and foot deformities ("mitten deformities"; pseudosyndactyly) (Figures 11 and 12) [58]. Two of these extracutaneous complications, esophageal strictures and pseudosyndactyly, are of particular importance, since they occur early in childhood and continue to negatively impact on the functionality of these patients throughout life. About 10% and 90% of all of these patients will develop symptomatic esophageal stricturing by ages 2 and 35, respectively [44]. Similarly, about 30% of severe generalized RDEB patients have signs of pseudosyndactyly as early as 2 years of age and virtually 100% will have developed this by age 20 [58].

Severe ankyloglossia and microstomia are the norm in severe generalized RDEB, and contribute to the markedly impaired oral intake of solid foods. Although secondary caries occur, no primary enamel defects exist in any type or subtype of dystrophic EB.

Chronic renal failure, the result of poststreptococcal glomerulonephritis or renal amyloidosis, occurs within this RDEB subtype, and may eventually lead to death in about 12% [59]. A low but real risk of potentially fatal dilated cardiomyopathy (4.5% cumulative risk by age 20, 30% of whom eventually die of this complication) exists in patients with severe generalized RDEB. Although the cause is unproven, data suggest the possibility that this may result from a micronutrient deficiency (carnitine; selenium) or chronic iron overload [60].

Although the risk of infantile death from any cause is low in RDEB, nearly all patients with severe generalized RDEB will develop at least one cutaneous squamous cell carcinoma (arising as early as within the second decade of life), and most (about 87% by age 45) will then die of metastatic squamous cell carcinoma within five years of the time of diagnosis of the first squamous cell carcinoma, despite apparent complete surgical removal of each primary carcinoma [32]. Rare children with severe generalized RDEB are also at risk of developing malignant melanoma (cumulative risk of 2.5% by age 12) although none of the latter has resulted in metastasis [32].

A more common RDEB subtype, formerly known as *non-Hallopeau-Siemens RDEB *(and probably best referred to as *generalized mitis RDEB*), has similar but less severe cutaneous involvement and a much lower risk of esophageal strictures, corneal injury, or hand or foot deformities. Growth retardation and anemia are extremely uncommon. However, these patients still have a significant risk of developing squamous cell carcinomas (47.5% by age 65), although the risk of death from metastases (60% by age 65) is lower than that which is seen in severe generalized RDEB [32].

A rare subtype of RDEB, *inverse RDEB*, is characterized by blistering and erosions which are primarily confined to intertriginous skin sites, the base of the neck, the uppermost back, and the lumbosacral area. Patients with this subtype are particularly prone to develop severe blistering within the oral cavity, esophagus, and the lowermost portion of the genitourinary tract. Debilitating strictures may eventually develop within the esophagus (cumulative risks of 10% and 90% by ages 5 and 30, respectively) [44] and vagina. These strictures may be extremely severe, markedly impairing intake of nutrients and normal sexual functionality. Although squamous cell carcinomas may also arise in these patients, the cumulative risk (23% by age 45) is much lower than that which is seen in either of the two generalized subtypes of RDEB [32].

Bullous dermolysis of the newborn may rarely be transmitted as a severe autosomal recessive disease. In the setting of this mode of transmission, it may prove fatal within early infancy. Other rare RDEB subtypes include pretibial RDEB, RDEB pruriginosa, and RDEB centripetalis [61], the latter of which is characterized by cutaneous disease activity which begins acrally and then progressively spreads toward the trunk over decades.

### Kindler syndrome

Kindler syndrome is characterized by generalized blistering at birth and the later development of characteristic poikilodermatous pigmentation and photosensitivity. Skin findings may include atrophic scarring and nail dystrophy, at times closely mimicking JEB-nH. Extracutaneous complications may include severe colitis, esophagitis, urethral strictures and, rarely, ectropions [3]. Teeth are uninvolved but gingival hyperplasia may develop. Skin derived squamous cell carcinomas have been reported in at least two of these patients.

## Etiopathogenesis [3]

### Genetics

Inherited EB is transmitted as either an autosomal dominant or autosomal recessive disease, depending on EB type and subtype. As noted elsewhere, most EB phenotypes have only one mode of genetic transmission. Of importance, spontaneous mutations for autosomal dominant disease are not uncommon in EBS but account for only the minority of cases of DEB lacking a known family history for the disease. Incomplete penetrance has been documented only rarely in autosomal dominant EB kindreds.

### Molecular basis of disease

Inherited EB has been shown to result from mutations in any of several structural proteins normally present within the keratinocyte or the skin basement membrane zone ("dermoepidermal junction") [3]. In general, the severity of skin and extracutaneous disease is a reflection of the type of mutation which is present, as well as the ultrastructural location of the targeted protein. Localized EBS, EBS-DM, and EBS generalized other (EBS-Koebner) result from dominant negative mutations within either the keratin 5 or keratin 14 gene. The site of mutation -- i.e., the location within the individual keratin filament -- is strongly correlated with EBS subtype, with EBS-DM patients having mutations within particularly structurally sensitive portions of the molecule. EBS with mottled pigmentation results from mutations within the keratin 5 gene, and EBS with muscular dystrophy is caused by mutations within the gene for plectin. A rare autosomal recessive form of EBS is known to result from mutations in the keratin 14 gene. EBS with pyloric atresia is caused by mutations either within the gene for plectin or the genes for the heterodimeric transmembrane protein, α6β4 integrin. Two suprabasal subtypes of EBS -- plakophilin deficiency [62] and lethal acantholytic EB [63] -- are known to arise as the result of mutations in the genes for plakophilin-1 and desmoplakin, respectively. The molecular etiology of EBS superficialis (EBSS) is still unclear although one family previously diagnosed with this was later shown to have a type VII collagen mutation similar to those seen in DDEB [64]. In support of EBSS being a distinctive entity, however, is the lack of any detectable type VII collagen mutation in the original published proband (unpublished data, 2009).

JEB-H results from severe mutations within any of the three genes which encode for the three-chained adhesion molecule, laminin-332 (previously named laminin-5). The presence of mutational hotspots with this disease facilitates rapid DNA screening in many patients. The majority of JEB-nH patients have less severe mutations within the same targeted genes, although a minority have mutations instead within the gene encoding for type XVII collagen (formerly known as bullous pemphigoid antigen 2 or BP-180). JEB with pyloric atresia is caused by mutations in either of the genes encoding for the two subunits of α6β4 integrin [65-67].

All types of DEB result from mutations within the type VII collagen gene (COL7A1) [68]. Of note, those with DDEB tend to have missense mutations resulting in glycine substitutions within the triple helical domain of type VII collagen. Analogous to what is seen in JEB-H and JEB-nH, patients with severe generalized RDEB may be either homozygous for their COL7A1 mutation or have two different mutations (i.e., "compound heterozygosity"), resulting in premature termination codons. In contrast, less severe types of mutations within the type VII collagen gene occur in patients with RDEB generalized other. In contrast to JEB, there are no mutational hotspots within the COL7A1 gene, so nearly every DEB family has its own unique site and/or type of mutation. A detailed summary of these mutations has been recently published [3].

Kindler syndrome, a rare autosomal recessive genodermatosis, is caused by mutations in the gene for kindlin-1, a recently discovered component of focal contacts in basal keratinocytes [69].

## Diagnosis/diagnostic criteria/diagnostic methods

### Diagnostic criteria

Each major EB type is diagnosed by determination of the ultrastructural level within which blisters develop following minor traction to the skin (Table 2). Subtypes are then defined on the basis of mode of transmission, immunohistochemical and electron microscopic findings, and clinical phenotype. Detailed summaries of the phenotypic features of each EB subtype have been recently published in an international consensus report on diagnosis and classification [3].

### Diagnostic methods

#### Postnatal diagnosis

In the absence of a well characterized proband within the same kindred, every patient suspected of having inherited EB should have one or more skin specimens harvested and properly processed for diagnostic immunofluorescence antigenic mapping (IAM) and transmission electron microscopy (TEM) [22]. The precise ultrastructural level of mechanical fragility and inducible blister formation can be ascertained by one or both of these diagnostic techniques. Details as to optimal harvesting of specimens and the transport solutions needed have been reviewed elsewhere [3,22]. In general, though, the best samples for IAM and TEM are small punch biopsies and shave biopsies, respectively, harvested from nonblistered skin which has been first subjected to mild rotary traction. The conventional inclusion of EB-relevant antibodies as part of the IAM study may allow further subclassification of these patients, based on the location, pattern, and relative intensity of staining by one or more of these antibodies. However, there is still sufficient overlap across some EB subtypes as to limit precise subclassification on every case based solely on these immunohistochemical findings even when additional monoclonal antibodies are employed [22,70,71]. TEM also permits the direct semi-quantification of specific ultrastructural structures (i.e., keratin filaments; hemidesmosomes; anchoring fibrils; subbasal dense plates). These findings can be helpful in subclassifying some cases. Of importance, when IAM and TEM results were compared on matched specimens harvested from a large number of consecutively biopsied EB patients, neither technique was proven to be more accurate, with a discordancy rate of only about 3%, suggesting that either technique, when properly processed and interpreted, will be equally informative diagnostically [22,72]. Given the technical difficulties associated with processing and interpreting EB specimens by these two techniques, non-molecular diagnostic studies on EB specimens are best done by a limited number of diagnostic reference laboratories having extensive experience in EB. A list of recommended laboratories may be found in the 2008 consensus report [3].

It is important to stress that routine histological processing of skin is not recommended in the setting of EB, since it may be difficult or impossible to distinguish at the light microscopy level between even lower intraepidermal and subepidermal cleavage in some specimens. Similarly, the precise distinction between intra-lamina lucida (i.e., JEB) and sub-lamina densa (i.e., DEB) cleavage can be ascertained only by IAM or TEM. As a further reason for not pursuing routine histology, most of the EB-relevant antibodies used in IAM cannot be employed on conventional formalin-preserved tissue samples, due to loss of antigenicity in the latter tissues. Alternatively, attempts at using these paraffin embedded tissue blocks for subsequent TEM evaluation are also invariably suboptimal, due to differences in the fixatives used for tissue preservation.

As discussed in the 2008 consensus report, DNA mutational analysis for subclassification of EB is still primarily reserved for prenatal diagnosis (and even then only when the causative mutation has already been identified in a proband within the same family), or when preimplantation therapy is being considered, given the considerable problems that have been seen with genotype-phenotype correlation within most of the EB subtypes [3]. In addition, DNA testing is not routinely performed in the absence of prior tissue confirmation of the major type of EB which is present, since there are too many genes potentially involved in EB to make simultaneous screening of multiple genes either practical or affordable. If and when gene replacement therapy becomes a reality, then DNA mutational analysis will become part of the work-up of all patients. Until that time, there may be other reasons to pursue DNA mutational analysis in selected cases. These include determination of the mode of transmission in patients suspected of having spontaneous mutations for DDEB (since the majority of these have been shown to have RDEB instead) or in patients involved in specific research projects which might benefit from full genotypic determination. Some families having one or more severely affected family members with autosomal recessive disease may also desire to screen clinically unaffected siblings for possible silent mutations, as well as their genetically unrelated spouses, prior to their pursuing pregnancy. Given how low the prevalence of autosomal recessive types of EB is within the general population, however, the likelihood of affected offspring arising from such pregnancies is extraordinarily low, making this very cost-ineffective.

### Prenatal diagnosis

Through the early 1990 s, prenatal diagnosis was routinely performed via either IAM or TEM on fetal skin specimens that were harvested, via ultrasound-directed fetoscopic sampling techniques, on or after about the 17 th gestational week [73,74]. Since the mid-1900 s, DNA mutational analysis has become the standard of care, using specimens obtained from chorionic villi, with the caveats noted above.

## Differential diagnosis

The size and validity of the differential diagnosis generated on a child or adult with blistering of the skin is truly a reflection of the level of training and expertise of the physician. Indeed, in most situations the diagnosis of inherited EB should be obvious to a dermatologist; in only a minority of cases will there be any need for a more extensive differential diagnosis to be entertained prior to tissue confirmation. In the neonatal period, however, *in utero *herpes simplex infection might need be considered, especially if there is no family history of a blistering disease or if the clinical findings are very atypical for EB. Other conditions that may be considered as part of the differential diagnosis of EB are summarized in Table 6[3].

**Table 6:** Differential diagnosis of inherited EB (in neonates and small children) *

Inherited or congenital disorders	
	Epidermolytic hyperkeratosis (bullous congenital ichthyosiform erythroderma)
	Ichthyosis bullosa of Siemens
	Peeling skin syndrome
	Pachyonychia congenita
	Congenital porphyrias
	Acrodermatitis enteropathica
	Incontinentia pigmentii
	Ectodermal dysplasia (ED)
	AEC syndrome (Hay-Wells syndrome)
	Congenital absence of skin (cutis aplasia)
	Congenital erosive dermatosis with reticulate supple scarring
	
Acquired disorders	
	
	Immunobullous disorders
	EB acquisita
	Linear IgA dermatosis
	Bullous pemphigoid
	Cicatricial pemphigoid
	Neonatal herpes gestationis
	Pemphigus
	
	Infectious diseases
	Herpes simplex
	Staphylococcal scalded skin syndrome
	Bullous impetigo
	
	Other diseases or conditions
	Bullous mastocytosis
	Traumatic blisters (sucking; other)

* modified from Fine JD et al, 2000 [5]

## Genetic counseling [75]

Genetic counseling is best performed either by a medical geneticist or a dermatologist who has considerable experience with EB. Ultimately the diagnosis will be based on clinical phenotype, mode of transmission, IAM, TEM, and, when available, the mutational analysis of the affected proband. In-depth recommendations on the counseling of EB patients and their families have been recently published [75].

## Management

The basic underlying tenets of care for all EB patients are avoidance of blistering (by meticulous protective padding of the skin) and prevention of secondary infection (by careful wound care, facilitated by the use of sterile synthetic non-adhesive hydrocolloid dressings). Patients with EB subtypes known to be at highest risk for specific extracutaneous complications need careful surveillance [76] for their occurrence, and implementation of appropriate interventions (medical; surgical; dental; nutritional; psychological; other) prior to the affected tissues becoming severely injured [24,25,76]. For example, early signs and symptoms of corneal disease activity need prompt evaluation by an ophthalmologist so as to prevent the development of permanent corneal scarring and impaired vision. Symptomatic esophageal strictures need to be dilated, oftentimes repeatedly, in order to maintain adequate intake of nutrients by mouth. Those children unable to take in sufficient nutrients by mouth are instead given nutrient supplements via gastrostomy [77]. Hand deformities, if they cannot be prevented by meticulous nightly wrapping of the digits, may be temporarily improved by surgical degloving procedures. Squamous cell carcinomas, which may arise as early as the second decade of life in patients with severe generalized RDEB and JEB-H, are treated by conventional wide excision, with careful follow-up to monitor for local or regional recurrence. Patients with generalized forms of JEB and RDEB need to be monitored by serial DEXA scans for possible osteoporosis or osteopenia, and in selected EB subsets other laboratory parameters (hematological; renal) or diagnostic tests (echocardiogram) should also be serially monitored or performed.

Several experimental approaches are now being explored for possible therapeutic use. These include, for autosomal recessive types of EB, *ex vivo *gene replacement [78,79], transplantation of allogeneic fibroblasts (in RDEB, to provide a source of normal type VII collagen) [80,81], transplantation of bone marrow-derived stem cells [82], and infusion of recombinant protein (i.e., type VII collagen for RDEB) [83]. For autosomal dominantly transmitted EB, a variety of studies are being pursued which are focused on means that possibly might either downregulate the dominant negative gene or, alternatively, compensate for its presence by the upregulation of other genes whose products might at least partially provide enhanced structural stability to the skin, thereby overriding the effect of the underlying mutation. Other clinical trials are now underway to look at possible means of enhancing wound healing, to include an ongoing one assessing the potential efficacy of the topical application of a small molecular weight protein, thymosin β4, to open wounds [84].

## Prognosis

The prognosis of EB is highly dependent on the subtype of disease that is present. Most EB patients, particularly those with EBS and DDEB, have normal life expectancies, but significant morbidity may complicate some. In contrast, patients with JEB, most notably those with JEB-H, are at major risk of death during the first few years of life, and patients with RDEB, particularly those with severe generalized RDEB, are at risk of death on or after young adulthood from metastatic squamous cell carcinoma.

## Unresolved questions

With rare exceptions, the underlying molecular cause of nearly every EB subtype has now been elucidated. Using such data, both postnatal and prenatal diagnoses are now possible for most clinical situations.

It is clear, however, that some patients with identical mutations may have vastly different clinical phenotypes from others, suggesting the likely presence of other factors that might contribute to such differences. Work is now underway in several laboratories to search for possible modifier genes which might contribute to the overall clinical severity within some of these EB subtypes and might better explain the range of clinical phenotypes which is observed within individual EB subtypes.

The remarkably high frequency with which squamous cell carcinomas arise in RDEB, as well as the risk of metastasis and death following wide surgical excision of the primary tumors, is essentially unique. Inexplicably these tumors act in a very biologically aggressive way despite being usually very well differentiated histologically. Several leading EB research groups, therefore, are now focusing their efforts on trying to determine why RDEB-associated squamous cell carcinomas differ so drastically in their behavior from those seen in the normal adult population, with the hope that a better understanding of the biology of these tumors in RDEB may result in more successful treatments or more effective means of prevention.

It is still unknown whether gene replacement will ultimately become a realistic therapeutic modality, although *in vitro *cell and *in vivo *animal studies continue to look promising. It is also as yet unknown whether stem cell transplantation, or implantation of viable allogeneic fibroblasts into the skin of patients with RDEB, might provide significant longterm clinical benefit and also be safely administered.

## Abbreviations

Epidermolysis bullosa: EB; EB simplex: EBS; EBS, Dowling-Meara: EBS-DM; EBS, Koebner: EBS-K; EBS, mottled pigmentation: EBS-MP; EBS superficialis: EBSS; Junctional EB: JEB; JEB, Herlitz: JEB-H; JEB, non-Herlitz: JEB-nH; Laryngo-onycho-cutaneous syndrome: LOC syndrome; Dystrophic EB: DEB; Dominant dystrophic EB: DDEB; Dystrophic EB, bullous dermolysis of the newborn: DEB-BDN; Recessive dystrophic EB: RDEB; RDEB, severe generalized: RDEB sev gen; RDEB, generalized other: RDEB gen oth; RDEB, inversa: RDEB-I; National EB Registry (USA): NEBR; Transmission electron microscopy: TEM; Immunofluorescence antigenic mapping: IAM;

## Competing interests

The author declares that he has no competing interests.
